# Anterior cervical spine surgery opens up concerns about thyroid function

**DOI:** 10.37796/2211-8039.1214

**Published:** 2021-09-01

**Authors:** Kaveh Haddadi, Saeed Heidarpour Khanghah, Siavash Moradi, Masoud Shayesteh Azar, Ozra Akha, Saeed Ehteshami

**Affiliations:** a Department of Neurosurgery, Orthopedics Research Center, Mazandaran University of Medical Sciences, Sari, Iran; b Department of Neurosurgery, Faculty of Medicine, Mazandaran University of Medical Sciences, Sari, Iran; c Education Development Center, Faculty of Medicine, Mazandaran University of Medical Sciences, Sari, Iran; d Department of Endocrinology, Diabetes Research Center, Faculty of Medicine, Mazandaran University of Medical Sciences, Sari, Iran

**Keywords:** ACDF, Anterior cervical, Discectomy and Fusion, Thyroid dysfunction

## Abstract

**Background and objectives:**

The anterior approach for cervical discectomy and fixation is a valuable procedure for decompression of the spinal cord in patients with severe canal stenosis and stabilization of cervical vertebral column. Although some studies have investigated the thyroid complications especially in cervical cancer surgery or recently in tracheostomy, little research has been performed on the anterior spine surgery so far. The present study aimed to evaluate possible changes in the thyroid in patients experiencing anterior cervical approaches for degenerative diseases.

**Materials and methods:**

Seventy patients who were undergoing anterior cervical spine surgery were selected and their demographic information was recorded, including age, sex, weight, body mass index (BMI), and medical records. Thyroid hormones (TSH, free T4, and free T3) were measured before surgery and three months after surgery.

**Results:**

Most patients had cervical disc herniation (60%). The mean duration of surgery was 71.9 ± 8.36 minutes (range: 60–90 minutes). Twenty-one patients (30%) had anterior plating while 49 patients (70%) did not. Spearman’s correlation coefficient was used to examine the correlation of the following variables with TSH changes: Number of operated cervical levels, level of operated spine, incision type, duration of surgery, type of surgery (ACDF or ACCF). None of these variables showed a significant correlation. Meanwhile, a significant and direct correlation was observed between TSH changes and age.

**Conclusions:**

Although the results of our study did not show any signs of functional changes due to thyroid tissue injury during surgery, based on rare case reports and age-related laboratory changes, we recommend thyroid function tests for diagnosing subclinical thyroid dysfunction before anterior cervical spine surgery in patients with degenerative diseases and especially in older adults.

## Introduction

The anterior approach for cervical discectomy and fixation has a long history since it was introduced by Smith and Robinson and Cloward [1–3].

Owing to its safety and value in the treatment of degenerative cervical disorders, anterior cervical discectomy and fusion (ACDF) is being better-received in cervical surgery with excellent clinical outcomes [4–6]. ACDF and anterior cervical corpectomy and fusion (ACCF) are two valuable procedures for decompression of the spinal cord in patients with severe canal stenosis and stabilization of cervical vertebral column [7–9]. ACDF is used when compression is limited to the disc level while ACCF is reserved for when the compression is extensive and involves vertebral body levels [7–10].

Recently, a rising number of studies have reported acceptable clinical and radiological outcomes following multi-level anterior cervical spine surgeries on ≥ 4 levels [11, 12].

In these anterior cervical approaches, surgeons need to be aware of the various potential complications. Complications may occur, as in any other procedure, from anesthesia to positioning, to exposure of dura mater and final instrumentation. However, reasonably few complications occur in anterior cervical spine surgeries [13].

In addition to significant complications like infections, wound hematoma, spinal cord or nerve root injury, anterior cervical spine surgery may entail prolonged retraction of anatomical structures such as the trachea, larynx, esophagus, and thyroid. Some of thyroid-related damages include thyroid nerve injury, traumatic damage, heat injury, and the long-term post-surgical compression of an instrument (cage or plate) [13–16].

Vakharia recently reported that hypothyroidism is a risk factor for poorer outcomes in patients undergoing primary ACDF. Therefore, proper preoperative improvement of thyroid hormones can minimize the incidence of developing numerous medical complications, in addition to decreasing complication rates and the total cost of treatment [17].

Although some studies have addressed thyroid complications especially in cervical cancer surgery or recently in tracheostomy procedures, little research has been performed on the anterior spine surgery.

A rise in the frequency of these surgeries in spine centers, possible complications of thyroid dysfunction after anterior cervical spine surgery, and the lack of relevant studies inspired us to prospectively study the patients with anterior cervical approaches for degenerative diseases to evaluate changes in the thyroid.

## Materials and methods

This prospective study enrolled patients who were candidates for ACDF or ACCF due to degenerative cervical spine diseases at the Neurosurgery Center of Imam Khomeini Hospital, Sari, Iran from March 2018 to April 2019.

### Protocol review

The study was approved by the local Ethics Committee (IR.MAZUMS.IMAMHOSPITAL. REC.1397.1401). Patients were briefed about the study objectives and procedures, and assured of the confidentiality of their data. The participants signed written informed consent forms. All the experimental procedures involving human samples were conducted with strict adherence to the guidelines of the declaration of Helsinki.

### Subjects

The participants were recruited over 1.5 years from among those who were admitted to our Neurosurgery Department for anterior cervical surgery due to degenerative diseases.

### Inclusion criteria

The inclusion criteria included being a candidate for ACDF or ACCF because of conditions such as cervical disc herniation, cervical spinal canal stenosis with anterior compression, and cervical myelopathy.

### Exclusion criteria

Patients with a history of cervical surgery, trauma, history of tracheostomy, radiotherapy of the neck, thyroid disorders, and history of exogenous thyroid hormones such as levothyroxine or anti-thyroid medications such as methimazole or propyl uracil, and history of a long hospitalization were excluded.

### Study design

The indication for surgery was confirmed by one spine neurosurgery fellow based on clinical signs and an MRI of the cervical spine. After entering the study, patients’ demographic information including age, sex, weight, body mass index (BMI), and medical history were recorded. All patients underwent a routine anterior cervical approach as introduced by Smith and Robinson. Type of cervical spine disease that warranted surgery, level of cervical spine involvement, and type of skin incision were also recorded. Thyroid hormones (TSH, free T4, and free T3) were measured before surgery and three months after surgery (to remove the effect of inflammation in releasing thyroid hormones). Additional significant complications were also evaluated and recorded. All laboratory tests were conducted in a blinded fashion.

### Outcome variables

The primary outcomes included motor and sensory scores upon admission, a full medical history, and a detailed physical and neurological examination. Laboratory examinations included complete blood count, routine serum electrolytes, glucose, urea nitrogen, and creatinine.

Thyroid hormones (TSH, free T4, and free T3) were measured before surgery and three months after surgery (to remove the effect of inflammation in releasing thyroid hormones).

Enzyme Linked Immunosorbent Assay (ELISA) was used for the quantitative measurement (Miu/ ml) of serum TSH, T4 and T3.

### Statistical analysis

Quantitative variables are presented as mean ± SD and qualitative variables as numbers (percentage, frequency, mean, minimum and maximum). First, normality of the data was investigated using Lilliefors-corrected *Kolmogorov*-*Smirnov* test. After confirming the normality of data, paired t-test was used to compare TSH, T3, and T4 before and after surgery.

Spearman’s correlation coefficient was used to assess the correlation between variables and TSH changes. Statistical analysis was performed in SPSS 25. P value < .05 was considered statistically significant.

## Results

In this study, 70 patients including 28 men (40%) and 42 women (60%) were examined. Their mean age was 43.87 ± 13.87 years (range: 20–73 years) with the highest frequency for the age group of 30–40 years (30%). Most patients had cervical disc herniation (60%). The mean duration of surgery was 71.9 ± 8.36 minutes (range: 60–90 minutes). Twenty-one patients (30%) had anterior plating and 49 patients (70%) did not. Table 1 shows the participants’ data. Furthermore, 45 patients (64.3%) had surgery at one level, 17 patients (24.3%) at two levels and 8 patients (11.4%) at three levels of cervical spine (Table 1).

Table 2 shows the mean (SD) of serum TSH, T3, and T4 levels before and after surgery (ng/ml), which are not significantly different.

**Fig 1:**
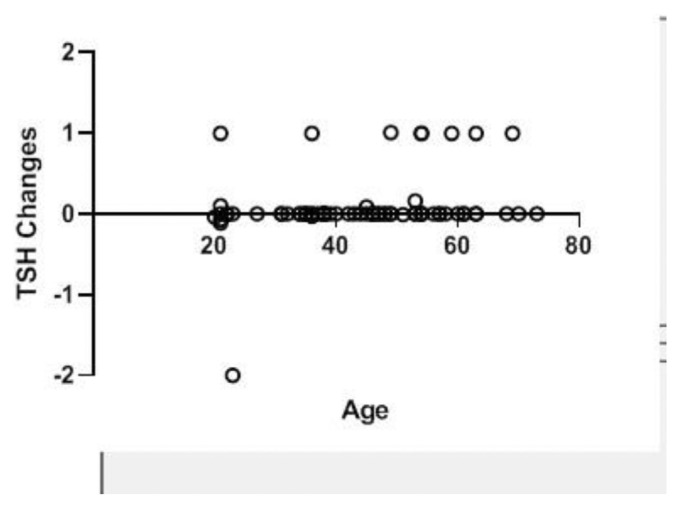
Correlation between TSH changes and age in patients undergoing anterior discectomy or corpectomy.

**Fig 2:**
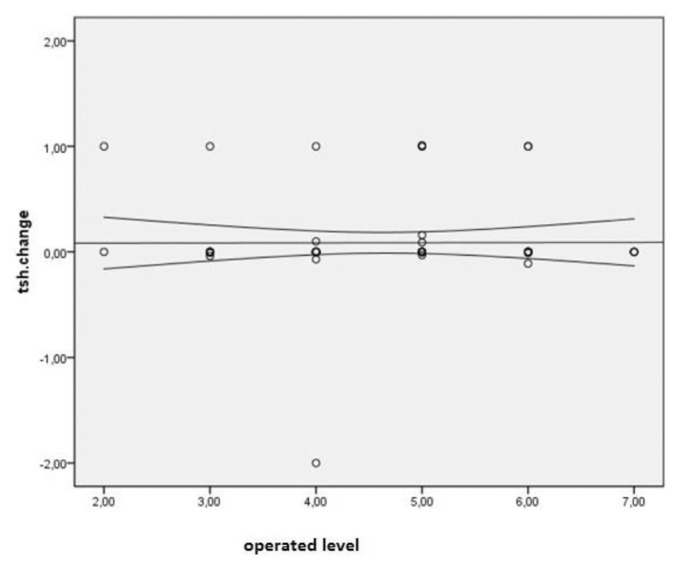
Distribution of correlation between surgical level and TSH changes in patients undergoing anterior discectomy or corpectomy.

**Fig 3:**
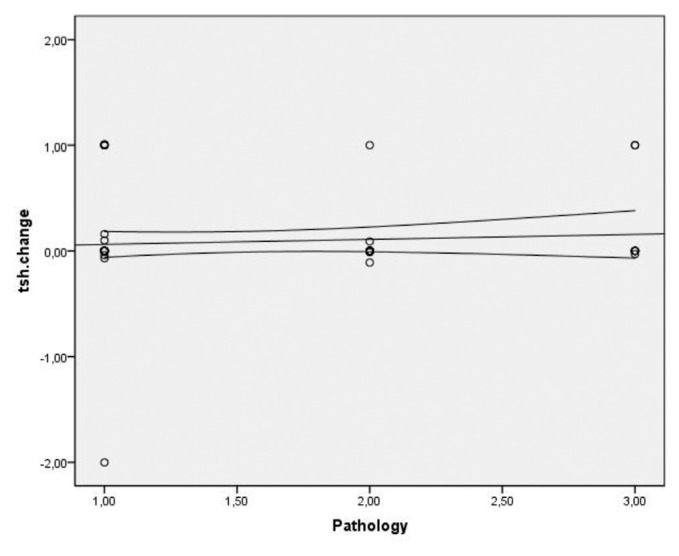
Distribution of correlation between pathology type and TSH changes in patients undergoing anterior discectomy or corpectomy.

**Fig 4:**
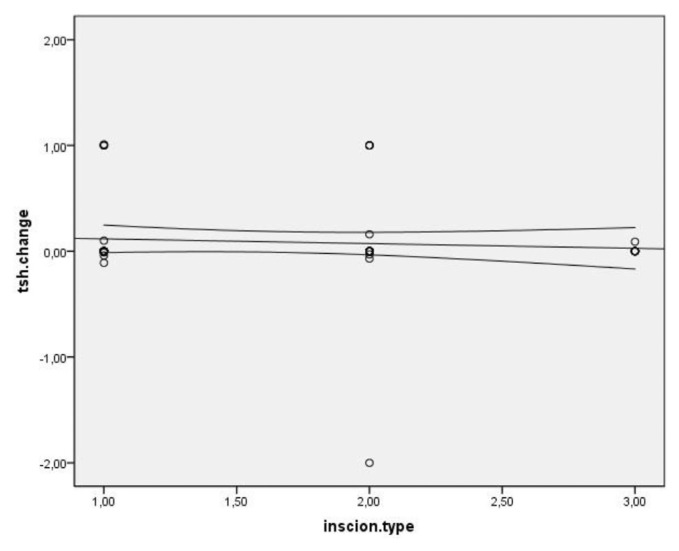
Distribution of correlation between type of incision and TSH changes in patients undergoing anterior discectomy or corpectomy.

**Fig 5:**
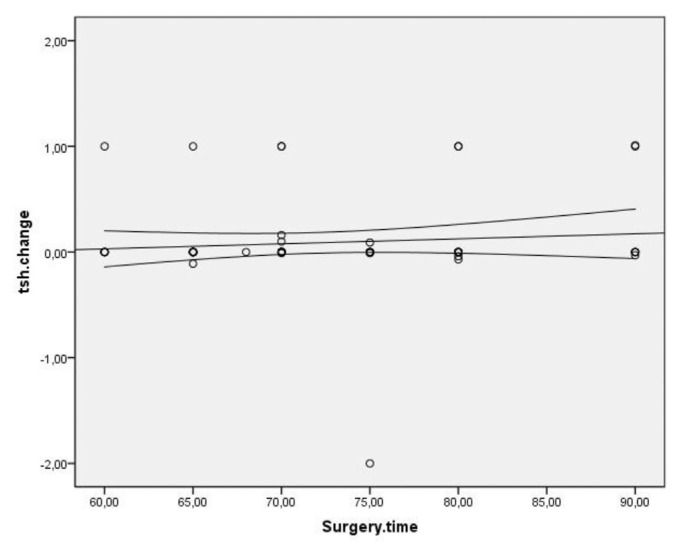
Distribution of surgical duration of correlation and TSH changes in patients undergoing anterior discectomy or corpectomy.

Spearman’s correlation coefficient was used to assess the correlation between TSH changes and the number of operated cervical levels, level of operated spine, incision type, duration of surgery, and type of surgery (ACDF or ACCF), which yielded no significant correlation. A significant and direct correlation was observed between TSH changes and age. That is, TSH changes increased with age (P = 0.007, r = 0.319) (Fig. 1). Other variables of the operated level (p = 0.002), pathology type (p = 0.053), type of incision (p = 0.042), anterior plating (p = 0.487), and duration of surgery (p = 0.008) did not show a statistically significant correlation with TSH changes (Figs. 2–5).

## Discussion

Cervical spine surgery comprises several common surgical techniques. The pathologies which need surgery include radiculopathy, myelopathy, instability caused by degeneration or trauma, infection, and tumors. Surgical plans comprise decompression of neural elements and stabilization, if required, via anterior, posterior, or combined approaches. The ideal approach is influenced by the site of the compression, type of injury, and the overall alignment [13].

Currently, ACDF and ACCF are generally recommended for patients with cervical radiculopathy refractory to conservative treatment with non-steroidal anti-inflammatory medications, physical therapy, or cervical corticosteroid injection. Nonetheless, the number of ACDF procedures is growing each year as the rate of hypothyroidism is [18–20].

However, surgeons need to be aware of the most important complications in anterior cervical surgery mentioned here: [21–29].

1. A wound hematoma, 2. Injury to the carotid or vertebral artery causing a stroke, 3. Bleeding, or even death, 4. Injury to the recurrent laryngeal nerve causing hoarseness, 5. Injury to the superior laryngeal nerve causing swallowing problem, 6.

Injury to the esophagus or trachea causing infection and mediastinitis, 7. Injury to the Dura mater causing a cerebrospinal fluid leak pseudomeningocele, 8. Mechanical complications of the graft and plate as well as graft migration, breakage of the plate, screw pullout, etc, 9. Wound infection, 10. Early or late painful pseudo-arthrosis due to failure of fusion and 11. Injury to the spinal cord or nerve roots causing sensory or motor neurologic dysfunction.

### Thyroid injury

The anterior cervical anatomy contains the thyroid gland and vital vessels in proximity of the operation site. Any intervention in this region can potentially influence the thyroid gland and consequently the metabolic cascade, cardiac circulation, and systemic circulation [30–32].

While considerable increases in thyroid hormone level can cause unexpected cardiac manifestations, particularly in patients with arrhythmic disorders, patients who undergo surgery in the anterior cervical region must be observed for a certain period after these procedures like tracheotomy [33–36].

Murat Karaman examined tracheostomy complication in Turkey, and reported that the mean free thyroxin and free triiodothyronine levels significantly increased from 0.08 ng/dL to 0.32 pmol/L in 20 patients who underwent tracheotomy due to respiratory problems three hours after the procedure as compared with before. In contrast, no such major rising was reported among persons who experienced percutaneous tracheostomy. The researchers declare that tracheotomy-induced rises in thyroid hormones should be seriously addressed [37].

In a retrospective cohort study by Vakharia et al. (2018), 90-day postoperative complications were examined in both hypothyroid and normal groups. They reported the odds of postoperative complications in hypothyroid patients were higher than those for euthyroid patients. They suggested that patients be screened for thyroid hormones before primary ACDF to reduce the side effects [17].

Huzurbazar et al. (2014) reported a case that developed hyperthyroid symptoms after C7 corpectomy. They stated that the patient had clinical signs of hyperthyroidism before surgery, but thyroid tests were not checked [38].

Here, we examined 70 individuals who needed anterior cervical surgeries due to degenerative diseases. Most surgeries were performed because of cervical disc herniation, and most of them received a single cervical level with a transverse incision without plating. Most patients had pathology at C5–C6 and C4–C5 levels. Mean levels of serum T3, T4, and TSH were normal before and after the surgery, and no thyroid dysfunction was observed in a three-month follow-up, but these results were not statistically significant. Our study also revealed a correlation between age and TSH changes before and after the surgery. It can be concluded that TSH changes increase with age. In our study, no patients developed clinical and subclinical thyroid malfunction or other related medical complications like new cardiac manifestations.

Additionally, various studies have suggested that trauma (such as cerebral palsy) and other acute stressing conditions cause hypermetabolic status due to cortisol secretion, and also change the thyroid hormone levels [39, 40].

Furthermore, a goiter (Enlarged thyroid gland) could increase the difficulty during exposure to anterior cervical spine approaches and could result in forceful retraction. Some authors advocated that Tailored approaches were necessary during the ACDF in order minimize the traction and thus the risk for complications. Surgeons should bear in mind that thyroid enlargement poses a risk during anterior spinal approaches and could cause injury on of the gland itself that among other can lead to postoperative dysfunction. In that case, a total or hemi- thyroidectomy provides a wide surgical field so the anterior cervical approaches, especially ACDF, are performed without exerting extreme traction, avoiding damage to adjacent neck structures, particularly the recurrent laryngeal nerve [41, 42].

Finally, the results of the present study show that ACDF surgery in our routine anterior surgery approach does not lead to thyroid disorders clinically or subclinically based on our clinical and laboratory policies, but long-term follow-up may be needed for a more accurate conclusion. We also found a significant change in TSH levels only with increasing age without any clinical effects on patient’s life. Also, no significant correlation was observed between other study variables like technique of operation, the pathology, the operated levels, the number of operated level, the time of operation, and the type of incision and serum thyroid hormone changes.

Ideally, chronic and permanent effects on thyroid gland via direct tissue damage or inflammatory processes should be followed in a long-term period after surgery and based on other diagnostic and laboratory measures like thyroid gland isotope scan in future.

## Conclusions

Although the results of our study did not show any signs of functional changes due to thyroid tissue injury during surgery, based on rare case reports and age-related laboratory changes, we recommend thyroid function tests for diagnosing subclinical thyroid dysfunction before anterior cervical spine surgery in patients with degenerative diseases and especially in older adults. However, more studies are necessary to specify the age group that thyroid function test are highly recommended during the pre- surgical work up of the patients that will undergo anterior cervical spine operations.

## Supplementary Information



## Figures and Tables

**Table 1:** Age ranges, type of pathology and other surgery information.

Variables		Distribution	Percent
Gender	Male	28	40
	Female	42	60
Age Range	20–30	19	27.1
	30–40	21	30.0
	40–50	16	22.9
	50–60	14	20.0
Pathology	Spinal stenosis	19	27.1
	Disc herniation	42	60
	OPLL	9	12.9
Number of operated levels	Single	45	64.3
	Double	17	24.3
	Triple	8	11.4
Operated level	C2–C3	2	2.9
	C3–C4	9	12.9
	C4–C5	21	30.0
	C5–C6	22	31.4
	C6–C7	12	17.1
	C7–T1	4	5.7
	Total	70	
Incision type	Oblique	49	70%
	Transverse	21	30%
Surgery time (min)	71.9 ± 8.36		
	Anterior plating	21	30
	Without anterior plating	49	70

**Table 2:** Mean differences (SD) of TSH, T3, and T4 before and after surgery (ng/ml).

Variables	Before Mean (SD)	After Mean (SD)	P-Value*
TSH	3.34 (0.98)	3.43 (1.02)	0.08
T3	1.35 (0.32)	1.29 (0.35)	0.079
T4	11.6 (8.22)	11.9 (8.17)	0.06

* Paired t-test.
