# Supplementary material for: Anterior cervical spine surgery opens up concerns about thyroid function
Source: Biomedicine (Taipei). 2021 Sep 1;11(3):31–7. doi: 10.37796/2211-8039.1214 (PMC8823493; doi:10.37796/2211-8039.1214)
Supplement: Supplementary file 1 [file bmed-11-03-031-s001.docx]

Hi dear editor of nice journal Biomedicine:

Thanks for your attentions

These are our responses to reviewer 2.

1. We add and highlights some grammatical corrections especially in abstract part.

2. We add some keywords and highlight them.

3. We rewrite the third paragraph of discussion part and highlight it.

4. We added some new illustration about goiter in anterior cervical surgery based two new requested references and highlight them in text.

5. We correct and highlight conclusion section.

Kings regard

Kaveh Haddadi
